# Genomic Investigation of Carbapenem-Resistant *Klebsiella pneumonia* Colonization in an Intensive Care Unit in South Africa

**DOI:** 10.3390/genes12070951

**Published:** 2021-06-22

**Authors:** Osama Madni, Daniel G. Amoako, Akebe Luther King Abia, Joan Rout, Sabiha Yusuf Essack

**Affiliations:** 1 Antimicrobial Research Unit, College of Health Sciences, University of KwaZulu-Natal, Durban 4000, South Africa; osama-merghani101@hotmail.com (O.M.); essacks@ukzn.ac.za (S.Y.E.); 2 School of Nursing, College of Health Sciences, University of KwaZulu-Natal, Durban 4000, South Africa; joanrout@worldonline.co.za

**Keywords:** carbapenem resistance, *Enterobacterales*, *Klebsiella pneumoniae*, whole genome sequencing, South Africa, intensive care

## Abstract

The study investigated carbapenemase-producing *Klebsiella* *pneumoniae* (CPKP) isolates of patients in an intensive care unit (ICU) in a public hospital in the KwaZulu-Natal province, South Africa using whole-genome sequencing (WGS). Ninety-seven rectal swabs, collected from all consenting adult patients (*n* = 31) on days 1, 3, and 7 and then weekly, were screened for carbapenemase-production using Chrome-ID selective media. Antibiotic susceptibility was determined for the fourteen positive CPKP isolates obtained using the VITEK 2 automated system. All isolates (100%) were resistant to ertapenem and meropenem, and 71.4% (*n* = 10) were resistant to imipenem. All CPKP isolates were subjected to ERIC/PCR, and a sub-sample of isolates was selected for WGS based on their antibiograms and clonality. All sequenced isolates harbored the *bla*_OXA-181_ carbapenemase (100%) and co-carried other β-lactamase genes such as *bla*_OXA-1_, *bla*_CTX-M-15_, *bla*_TEM-1B,_ and *bla*_SHV-1_. IncF, IncX3, and Col plasmid replicons groups and class I integrons (ln191 and ln27) were detected. All isolates belonged to the same sequence type ST307 and capsular serotypes (K102, O2v2). All the isolates carried the same virulence repertoire, reflecting the epidemiological relationship between isolates. *bla*_OXA-181_ was located on a multi-replicon plasmid similar to that of *E. coli* p010_B-OXA181, and isolates were aligned with several South African and international clades, demonstrating horizontal and vertical transboundary distribution. The findings suggest that *bla*_OXA-181_ producing *K. pneumoniae* is endemic in this ICU, colonizing the patients. CRE screening and enhanced infection prevention and control measures are urgently required.

## 1. Introduction

β-lactams are one of the most prescribed antibiotics globally [1], in part because they are flexible and can be structurally modified to result in several active forms, a malleability not possessed by other antibiotic classes [2]. Carbapenems, among the β-lactams, are effective against Gram-positive and Gram-negative bacteria, providing a broad spectrum of antibacterial activity. A particular molecular structure, along with the β-lactam ring, gives added stability to most β-lactamases, including extended-spectrum β-lactamases (ESBLs), making carbapenems “last-resort antibiotics” or “last-line agents” as treatments for critically ill patients suspected of drug-resistant infections [3]. The widespread use of carbapenems for multidrug-resistant bacterial infections has contributed to the development of resistance, most frequently mediated by β-lactamases, called carbapenemases [4]. Less common resistance mechanisms include altered target penicillin-binding proteins (PBPs) or reduced cell entry due to upregulated efflux pumps or modifications in the outer membrane proteins [5].

Carbapenemases belong to the Ambler class A (e.g., KPC, SME, IMI, and GES), class B (e.g., NDM, VIM, and IMP), and class D (OXA-48–like). The carbapenemases of class A and class D are serine β-lactamases, and the carbapenemases of class B are metallo-β-lactamases (MBLs) [6]. Class B carbapenemases, particularly NDM, are more potent than other classes and cannot be inhibited by β-lactamase inhibitors (BLIs) such as clavulanic acid, tazobactam, or sulbactam [7]. OXA carbapenemases, and specifically *bla*_OXA-48_, have a more restricted spectrum of β-lactam hydrolysis with clinically significant penicillin and imipenem hydrolysis and lower meropenem hydrolysis [5]. BLIs poorly inhibit OXA carbapenemases except for vaborbactam [8]. The *bla*_OXA-181_ gene, being a variant of *bla*_OXA-48_, was identified in different members of the *Enterobacterales* [9,10].

Carbapenem-resistant *Enterobacterales* (CRE), *Pseudomonas aeruginosa,* and *Acinetobacter baumannii* are listed by the World Health Organization (WHO) as high-priority pathogens for the research and development of new antibiotics [11]. Severe CRE infections have a higher risk of fatality than those caused by carbapenem-sensitive isolates [12]. This threat is considerably higher when the carbapenem resistance in *Enterobacterales* is due to carbapenemase production (CPEs; carbapenemase-producing *Enterobacterales*). Compared with the non-CPE infections (*n* = 37,45%), CPE infections (*n* = 46, 55%) were reported to cause four times the risk of death within 14 days among hospitalized patients in an academic hospital in the USA between March 2013 and April 2016 [13].

Carbapenemase resistance genes are associated with diverse mobile genetic elements (MGEs), including but not limited to plasmids, transposons, and integrons [14]. IncF plasmid replicons are the most common and are primarily reported to bear the *bla_KPC_* and *bla_NDM_* genes [15]. The transposon Tn*125*, harboring the insertion element IS*Aba125*, is recognized as the primary vehicle for disseminating *bla*_NDM-1_ genes in *Acinetobacter baumannii* [16]. The successful propagation of *bla*_OXA-48_ is linked with different Tn*1999* variants on highly transferable IncL plasmids, strengthened by the global distribution of specific high-risk clones such as *Klebsiella pneumoniae* ST307 [17].

*Klebsiella pneumoniae* is an invasive and virulent bacterium among *Enterobacterales* harboring multiple antibiotic-resistance genes (ARGs) [18]. *K. pneumoniae* easily assimilates ARGS by horizontal gene transfer (HGT) of MGEs [19]. The increase in multi-drug-resistant (MDR) *K. pneumoniae* and especially carbapenem-resistant *K. pneumoniae* (CRKP) is of significant medical concern. A recent meta-analysis approximated mortality associated with *K. pneumoniae* healthcare-associated infections (HAI) at 42% for CRKP compared with 21% for carbapenem-susceptible strains [20]. We used whole-genome sequencing (WGS) and bioinformatics analysis to characterize CPE implicated in the colonization of patients in an intensive care unit (ICU) of a public hospital in the uMgungundlovu District KwaZulu-Natal, South Africa.

## 2. Materials and Methods

### 2.1. Study Population

The study focused on the incidence and transmission of CPEs among ICU patients receiving carbapenem treatment at the uMgungundlovu District hospital from February 2020 to March 2020. This study’s participants were all consenting adult patients admitted over one month into the general ICU (medical and surgical).

### 2.2. Sample Collection and Screening for CPEs

Patients were screened for CPE using rectal swabs on days 1, 3, and 7 and weekly after that until transfer (to another ward or healthcare institution), discharge, or death.

Ninety-seven rectal swabs from 31 patients were screened for CPE using chromogenic screening media Chrom-ID CARBA SMART according to manufacturer instructions (BioMérieux, Marcy l’Étoile, France). Fourteen samples, positive for CPEs (14.4% of total samples) from five patients (16% of total patients enrolled), formed the final sample.

### 2.3. Antimicrobial Susceptibility Testing (AST)

Identification of the isolates to species levels and antibiotic susceptibility testing was conducted using the VITEK 2^®^ automated system (BioMérieux-Vitek, Marcy-l’Étoile, France), and results were interpreted according to the European Committee on Antimicrobial Susceptibility Testing (EUCAST) breakpoints. The antibiotic panel consisted of 18 antibiotics: ampicillin (AMP), amoxicillin-clavulanic acid (AMC), piperacillin-tazobactam (TZP), cefuroxime (CXM), cefuroxime-axetil (CXM-AX), cefoxitin (FOX), cefotaxime (CTX), ceftazidime (CAZ), cefepime (FEP), ertapenem (ERT), imipenem (IPM), meropenem (MEM), amikacin (AMK), gentamicin (GEN), ciprofloxacin (CIP), tigecycline (TGC), nitrofurantoin (NIT), and trimethoprim-sulfamethoxazole (SXT).

### 2.4. Genomic Characterization

#### 2.4.1. DNA Extraction of CPKP Isolates

Genomic DNA was extracted using GenElute bacterial genomic DNA kit (Sigma-Aldrich, St. Louis, MO, USA) according to the manufacturer’s instructions for further molecular tests. Extracted DNA was checked for suitable concentrations and purity using Nanodrop 8000 spectrophotometer (Thermo Scientific, Waltham, MA, USA).

#### 2.4.2. Genomic Fingerprinting

Clonality was investigated using Enterobacterial repetitive intergenic consensus-PCR (ERIC-PCR). The primers ERIC1 (5′-ATGTAAGCTCCTGGGATTCAC-3′) and ERIC2 (5′-AAGTAAGTGACTGGGTGAGCG-3′) (Inqaba Biotech (Pty.) Ltd., Pretoria, South Africa) were used to perform ERIC-PCR reactions on a T100 Thermal Cycler (Bio-Rad, Hercules, CA, USA) with a total volume of 25 μL consisting of 12.5 μL of Dream Taq Green PCR Master Mix (2X) (ThermoFisher Scientific, Waltham, MA, USA), 0.1 μL (100 μM) each of primers ERIC1 and ERIC2, 8.3 μL of nuclease-free H_2_O, and 4 μL of total genomic DNA. The PCR conditions were initial denaturation at 94 °C for 3 min, followed by 29 cycles denaturation at 94 °C (30 s), annealing at 50 °C (1 min), an extension at 65 °C (8 min), and a final extension at 65 °C (16 min). PCR amplicons were resolved horizontally by electrophoresis on 1.5% (*w*/*v*) on SeaKem^®^ LE agarose gel at 90 V for two hours (Lonza, Rockland, ME, USA) followed by staining in 0.1 mg/mL of ethidium bromide for 15 min. The Quick-load^®^1-kb DNA ladder (New England BioLabs, Ipswich, MA, USA), a positive control (*K. pneumoniae* ATCC BAA-1705), and no-template control (NTC) were included in these reactions. The gel was visualized, and the images were captured under ultraviolet light using the Gel Doc^TM^ XR+ system (Bio-Rad, Hercules, CA, USA). Data was exported to BioNumerics software (version 7.6, Applied Maths, Austin, TX, USA) for cluster analysis. Strains were allocated to various clusters by evaluating the similarity coefficient from the homology matrix using the Jaccard approach. Dendrograms were assembled using the Unweighted Pair-Group Method (UPGMA). A 1% optimization and band tolerance were set, and 90% cut-off similarity was used to identify clusters.

#### 2.4.3. Whole Genome Sequencing (WGS) of CPKP Isolates

A sub-sample of ten isolates, one per patient, were chosen based on similar antibiograms and ERIC profiles and subjected to whole-genome sequencing on the Illumina MiSeq platform. Using the Nextera XT DNA sample preparation kit (Illumina, San Diego, CA, USA), multiplexed paired-end libraries (2300 bp) were prepared, and sequences were measured on 100-coverage. Utilizing CLC Genomics Workbench version 10, the resulting raw reads were screened for quality, trimmed, and de novo assembled into contigs (CLC, Bio-QIAGEN, Aarhus, Denmark). Two isolates were removed due to their poor DNA quality, and the remaining eight isolates were used for further downstream analysis.

#### 2.4.4. Genomic Analyses and Annotation

The National Centre for Biotechnology Information (NCBI) prokaryotic genome annotation pipeline (PGAP) and RAST 2.0 server [21] were used to annotate assembled reads. All contiguous sequences were subsequently submitted to GenBank and assigned accession numbers under the Bioproject (PRJNA674742). ARGs were detected using ResFinder [22], and plasmids were delineated using PlasmidFinder [23]. The RAST SEED viewer [24] was employed to detect the genomic characteristics of the contigs and the presence of transposases flanking the β-lactamase genes (or carbapenemases). The INTEGRALL database was used to assess integrons (http://integrall.bio.ua.pt/). Insertion sequences (IS) residing in genomes were inferred by uploading contigs to the MobileElementFinder [25]. The BacWGSTdb and Pathogenwatch servers were employed to infer virulence genes and factors (http://bacdb.cn/BacWGSTdb/, https://pathogen.watch/ (accessed on 17 February 2021)). Kaptive-web, the reference online site tool for *Klebsiella* WGS data, was used to predict isolates’ serotypes (K types, wzc, and wzi allelic types, and O types) [26]. Multilocus sequence type (MLST) analyses were performed to determine sequence types (STs) of the study isolates on the PubMLST database (https://pubmlst.org/).

The carbapenemase genes and their flanking sequences retrieved from the RAST SEED viewer were searched on the NCBI microbial nucleotide basic local alignment search tool (BLAST). Fully sequenced plasmids with the closest synteny obtained from the BLAST search were used as a reference input to GView Server (https://server.gview.ca/) to visualize the presumed presence/absence of specific plasmid DNA.

#### 2.4.5. Phylogenomics and Epidemiological Insights

Phylogenomic analysis was undertaken to determine how the study isolates compare to *K. pneumoniae* genomes from South Africa. All *K. pneumoniae* genomes reported in South Africa (*n* = 89) were downloaded from the PATRIC website (https://www.patricbrc.org/), annotated (Table S1), and included in the analysis. The phylogenetic tree was constructed based on the maximum likelihood method using the CSIPhylogeny (https://cge.cbs.dtu.dk/services/CSIPhylogeny/ (accessed on 23 February 2021)) [27], which performs single-nucleotide polymorphism (SNP) calling, filters the SNPs, and infers phylogeny based on the concatenated alignment of the high-quality SNPs, using the assembled contigs. The analysis was performed on the platform using default parameters as follows: minimum depth at SNP positions, 10X; minimum relative depth at SNP positions of 10%; minimum distance between SNPs (prune) at 10 bp; minimum SNP quality, 30; minimum read mapping quality of 25, and minimum Z-score of 1.96. The *Escherichia coli* ATCC 25922 was used as the outgroup strain (reference genome), facilitating the configuration of the phylogenetic distance between the isolates on the branches. The Figtree software (http://tree.bio.ed.ac.uk/software/figtree/ (accessed on 23 February 2021)) was used to visualize, edit, and annotate the generated phylogenetic tree.

## 3. Results

### 3.1. Population Demographics

Of the five consenting adult patients who screened positive for CPE, three were males, while two were females, aged between 26 and 64. Although they were admitted to the ICU with different diagnoses, almost all had the same invasive devices. The relevant patients’ data appear in Supplementary Table S2.

### 3.2. Antimicrobial Resistance Profiles

**Figure 1:**
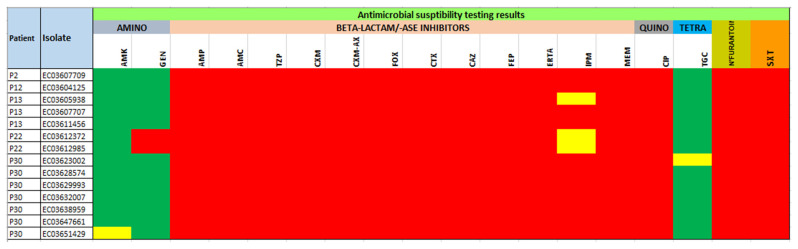
Antibiotic susceptibility patterns of CPKP isolates (*n* = 14). Antibiotic susceptibility profiles are shown for the fourteen sequenced *K. pneumonia* isolates. Antibiotic resistance is displayed in red, intermediate resistance is displayed in yellow, and susceptibility is displayed in green.

Isolates showed a similar resistance pattern to the panel of antibiotics. All isolates (100%) were resistant to 14 antibiotics, viz., ampicillin, amoxicillin-clavulanic acid, piperacillin-tazobactam, cefuroxime, cefuroxime-axetil, cefoxitin, cefotaxime, ceftazidime, cefepime, ertapenem, meropenem, ciprofloxacin, nitrofurantoin, and trimethoprim-sulfamethoxazole. However, they were fully susceptible to tigecycline and amikacin. Twelve (85.7%) isolates were susceptible to gentamycin, and four (28.6%) were susceptible to imipenem. All isolates were MDR, and the resistance phenotype was confirmed by the ARGs detected by WGS (Figure 1 and Table S3).

### 3.3. Genomic Fingerprinting

**Figure 2:**
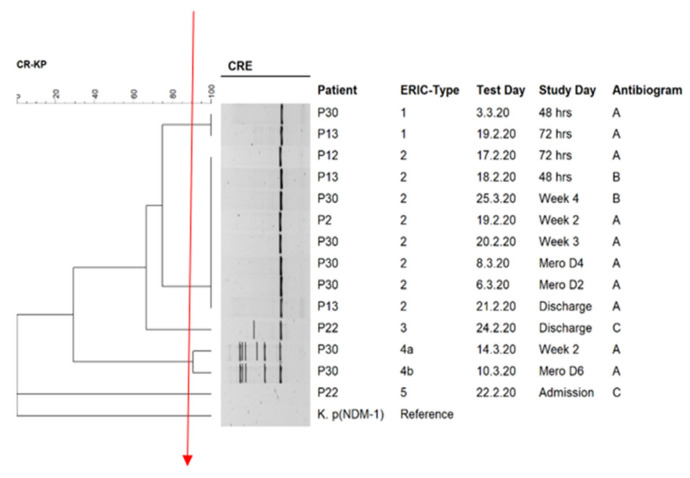
ERIC/PCR dendrogram for CPKP isolates (*n* = 14). Isolates clustered into five main clusters based on the similarity index of 90%. The solid red line indicates the ERIC-type cut-off. Antibiogram: A = AMP-AMC-TZP-CXM-CXM-AX-FOX-CTX-CAZ-FEP-ERTA-IPM-MEM-CIP-NIT-SXT; B = AMP-AMC-TZP-CXM-CXM-AX-FOX-CTX-CAZ-FEP-ERTA-MEM-CIP-NIT-SXT, C = AMP-AMC-TZP-CXM-CXM-AX-FOX-CTX-CAZ-FEP-ERTA-MEM-GEN-CIP-NIT-SXT; Mero D6 = 6th day of meropenem therapy; Mero D = 4th day of meropenem therapy and Mero D2 = 4th day of meropenem therapy.

The ERIC dendrogram showed five main clusters. Isolates belonging to the same ERIC cluster generally had similar antibiograms, except for ERIC cluster 2, where isolates exhibited two antibiograms. Patients generating more than one CPE sample carried isolates belonging to 2–4 different ERIC clusters (e.g., isolates 13, 22, and 30) (Figure 2 and Table 1). All the isolates belonged to ST307 and harbored the *bla*_OXA-181_ carbapenemase and different combinations of other β-lactamases (Table 1).

### 3.4. Genome Characteristics of the Isolates

The draft genomes sizes of the eight isolates subjected to WGS and bioinformatics analysis were similar and ranged from 5.58 Mb to 5.72 Mb. The (G + C) content was similar for all isolates (57.1) except two that differed slightly in terms of median contigs lengths (N50, L50), contigs, RNAs present, and the number of coding sequences (Table S4).

### 3.5. In Silico ARGs Analysis

The genomic data (resistomes) confirmed the resistance phenotypes in most isolates (Table 1). Anomalies were detected for aminoglycosides where the presence of the *aadA1* (*n* = 2), *aadA2* (*n* = 1), *aac(6′)-Ib-cr* (*n* = 8), *aph(3″)-Ib* (*n* = 5), *aph(3′)-Ia* (*n* = 2), *aph(3′)-VIa* (*n* = 2), *armA* (*n* = 2), *aph(6)-Id* (*n* = 5), and *aac(3)-Ia* (*n* = 1) aminoglycoside ARGs did not translate into phenotypic aminoglycoside resistance on AST (Table S3). A single carbapenemase gene (*bla*_OXA-181_) was common to all isolates. Five other β-lactamase were detected (*bla_CTX-M-15_*, *bla_OXA-1,_ bla_SHV-106_*) (*n* = 8), *bla_TEM-1B_* (*n* = 7), and *bla_TEM-1C_* (*n* = 1). Carbapenemases from classes A, B, and C were not detected. Other ARGs detected conferred resistance to tetracyclines (*tet(A)*) (*n* = 7), fluoroquinolones (*aac(6′)-lb-cr*, *oqxA*, *oqxB*, *qnrB1*, *qnrS1*) (*n* = 8), trimethoprim (*dfrA12* (*n* = 1), *dfrA14* (*n* = 7)), sulphonamides (*sul1* (*n* = 1), *sul2* (*n* = 5)), fosfomycin (*fosA*) (*n* = 8), macrolides (*msr(E)* (*n* = 2), *mph(E)* (*n* = 2)), phenicols (*catB3* (*n* = 8), *cmlA1* (*n* = 2)), rifamycin (*ARR-2*) (*n* = 1) and streptogramin b (*msr(E)* (*n* = 2)).

### 3.6. Sequence Types Analysis (MLST), In Silico Mobile Genetic Elements (Mobilome), Virulome and Capsular Serotypes

**Figure 3:**
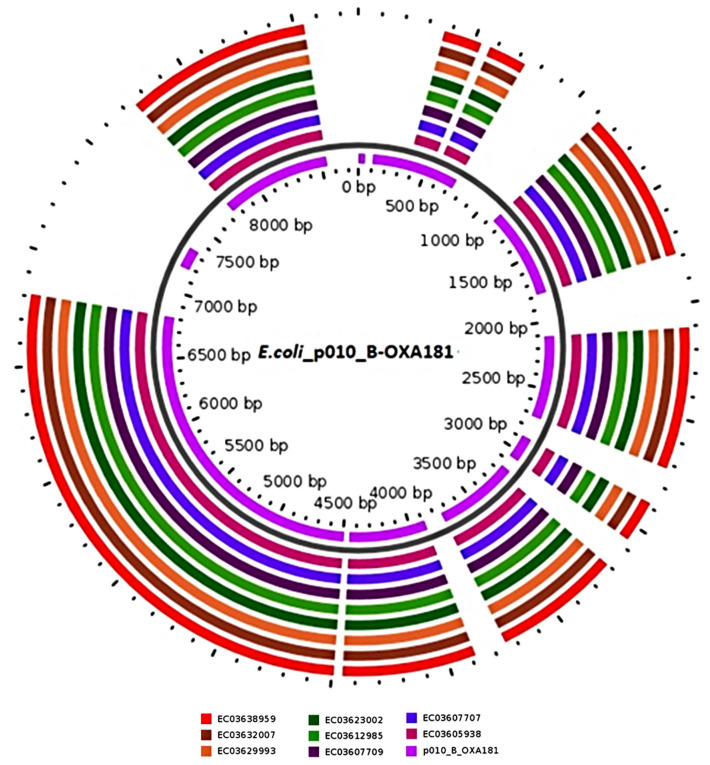
Comparison of *E. coli* plasmid p010 B-OXA181 with genome assemblies containing OXA-181 in CPKP isolates (*n* = 8). The map was developed using the online server GView (https://server.gview.ca/. Accessed on 24 February2021). The concentric circles reflect genome assemblies containing OXA-181 in *K. pneumoniae* isolates for comparisons with *E. coli* p010-B-OXA181, starting with the inner circle. Color codes are assigned for every isolate with a plasmid sequence identity, ranging from 99–100%.

All isolates belonged to ST307 (Table 1). Plasmid analysis showed that each isolate harbored at least two replicon types simultaneously (Table 2). All isolates carried ColKP3 and 6, 7, and six isolates carried the IncX3, IncFIB (K), and IncFII (K) replicons, respectively. One isolate carried the IncFIB (pNDM-Mar) from the same group. The search for carbapenemase (OXA-181) and its flanking sequences using NCBI microbial nucleotide BLAST showed that it was located on a multi replicon plasmid *E. coli* p010-B-OXA181-like (accession no.: CP048332.1) in the CPKP isolates. Comparative analyses confirmed the presence of genome assemblies with similar genetic synteny and 99.98% coverage and identity to the *E. coli* p010 B-OXA181-like reference through the GView server (Figure 3) intimating the location of OXA-181 on a similar plasmid in all the CPKP isolates.

The determination of the location of ARGs and MGEs in synteny in the study isolates on plasmids and chromosomes on the NCBI databases revealed their location on the plasmids except for one β-lactamase gene, *bla*_SHV-106_, that was encoded on a chromosome (Table 3). It is noteworthy that the IS*Kpn19* insertion sequence containing the *bla*_OXA-181_ also harbored the *EreA* and *QnrS1* genes in seven of the eight isolates together with recombinase and transposases. One isolate carried the same insertion sequence but was located on transposon (Tn*3-like* IS*3000* transposase), found in the same plasmid and with the same companions (Table 3).

We further identified *bla*_SHV-106_ on all isolates in parallel with other β-lactamase genes viz., *bla*_OXA-1_, *bla*_CTX-M-15_, *bla*_TEM-1B_, and *bla*_TEM-1C_ in various permutations and combinations, the synteny of which was closely related to plasmid sequences already described in Genbank and summarized in Table 3. Across the isolates, *bla*_CTX-M-15_ (bracketed by Tn*3* transposon, IS*6100* and IS*Ec9*); most *bla*_TEM-1B_ genes (bracketed by IS*91*, and a recombinase); and most *aph(6)-Id*, *aph(3”)-Ib*, *sul2* genes (bracketed by IS*91*, IS*5070*, and IS*5*) were found in close synteny on identical plasmids, except for one isolate (EC03612985) that encoded both *bla_CTX-M-1_* and *bla_TEM-1C_* on one insertion sequence (IS*Ec9*), showing a slightly different genetic environment (Table 3).

All isolates had trimethoprim and aminoglycoside resistance genes located on a class 1 integron (In191), with five isolates also containing the *dfrA14* genes on gene cassettes. Another class 1 integron (In*27*) was detected in only one isolate and contained the *dfrA12-gcuF-aadA2* cassette arrays. Only one isolate did not have any integrons with aminoglycosides or trimethoprim-resistance genes (Table 2).

All isolates carried an identical repertoire of 65 virulence genes and hence were clonal (Table S5). The virulence determinants belonged to the eight major virulence factor classes of Klebsiella: adherence, biofilm formation, efflux, immune evasion, iron uptake, nutritional factor, secretion system, and toxin. The virulence The O and K capsules (KL102 and O2v2) types in the strains were also highly clone specific.

### 3.7. Phylogenomic Analysis

**Figure 4:**
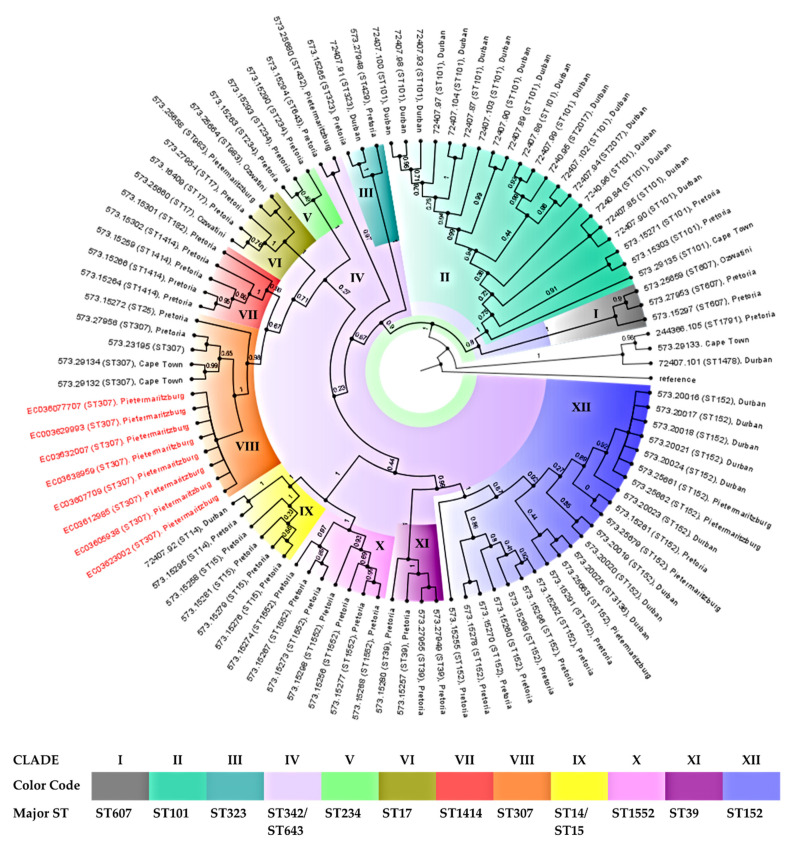
Phylogeography and resistance profiles of *K. pneumoniae*. The phylogeographic relationship between the eight *K. pneumoniae* isolates with other South African *K. pneumoniae* isolates. The *Escherichia coli* ATCC 25922 was used as the outgroup strain (reference genome).

Although all eight isolates belonged to the same sequence type, ST307, small differences were evident from their phylogenetic tree (Figure 4). When compared to genomes of *K. pneumoniae* isolates from studies conducted in Durban, Pretoria, Pietermaritzburg, Ozwatini, and Cape Town (Table S1), the study isolates showed the greatest similarity to the clade of ST307 from Pretoria and Cape Town (clade VIII) with one ST25 clustered close to the ST307 clade (Figure 4). Of note, even though the isolates clustered according to their sequences, whole-genome phylogenetics showed higher resolution than the MLST typing scheme.

## 4. Discussion

The global spread of CPE presents a significant challenge to public health and clinical practice as these bacteria are resistant to last-resort antibiotics (carbapenems) and cause high fatality rates [28]. WGS data for eight CPKP strains isolated from patients at a general ICU of a regional hospital in South Africa were analyzed to delineate the genetic context of carbapenem resistome and associated mobilome together with the broader resistome virulome, clonality, and phylogeny.

*K. pneumoniae* is an established and significant pathogen in nosocomial infections. MDR and extremely drug-resistant (XDR) *K. pneumoniae* are closely linked to ARGs acquired via plasmids and other MGEs, resulting in a “super resistome” [29]. CRKP has been implicated in outbreaks in South Africa and appears to be endemic to certain hospitals and regions. In May 2012, a tertiary academic hospital in Cape Town reported one of the first laboratory-confirmed outbreaks of *K. pneumoniae* expressing OXA-181 in South Africa, linked to two patients admitted to the hematology ICU. Before then, laboratory records and the results of 340 rectal swabs or stool specimens collected from patients and staff members revealed an “outbreak” involving eight patients. Seven of them were admitted to the hematology unit during their hospitalization period. The remaining patient was epidemiologically linked to another patient because he was admitted to an adjacent bed [30].

The identification of ARGs from WGS data correlated with the phenotypic resistance observed in most instances in the study, with resistance ranging from 14% to 100%. However, some anomalies were observed. For example, the presence of aminoglycoside ARGs on WGS was not phenotypically evident, intimating transcriptionally silent or unexpressed ARGs. More so, the discrepancy between the aminoglycoside-modifying enzymes and phenotypes is not peculiar and has been widely reported in Carbapenemase- and ESBL-producing *Enterobacter* species [31,32,33]. This necessitates the urgent development of phenotypic assays that can accurately and rapidly predict aminoglycoside responses among Carbapenemase and ESBL-producing *Enterobacter* spp. to avoid treatment failures. All isolates belonged to the same ST type and the same phylogenetic clade, suggesting an endemic strain in the ICU.

The study findings showed that the CPKP hospital population was not diverse and belonged to a single sequence type (ST307) (Table 1), clade, and capsular serotype and contained identical repertoires of virulence genes. The phylogeography analysis with South African *K. pneumonia* isolates showed the isolates clustering in clade VIII to be similar to isolates from Pretoria and Cape Town (Figure 4). The Pretoria study characterized 56 CRKP with reduced susceptibility to carbapenems from six public hospitals and centers in the Tshwane District in 2018. The *K. pneumoniae* isolates belonged to ST307 and had similar resistance determinants, particularly *bla*_OXA-181_, along with *bla*_OXA-1_, *bla*_TEM-1B_, *bla*_CTX-M-15_, and *bla*_SHV-28_ genes. The similar plasmid (Inc) types (ColKP3, IncX3, IncFIB (k), IncFII (k)) and serotypes (KL102, O2v2) were evident [34], suggesting that the same strain is circulating in the Kwazulu-Natal area. More so, observation of imipenem-susceptible, meropenem-resistant *Klebsiella pneumoniae* producing OXA-181 was not peculiar, as it has been reported in the literature [35].

*Klebsiella pneumoniae* ST307 is a relative newcomer to successful high-risk antibiotic-resistant strains contributing to a significant global public health burden. It possibly originated in Europe during the early to mid-1990s but was first published in the MLST database from The Netherlands in 2008 (https://bigsdb.pasteur.fr/klebsiella/klebsiella.html. Accessed on 24 February2021). The earliest published reports came from the USA [36] and Pakistan [37] in 2013 and were followed by reports from different countries, including Russia [38], Spain [39], Brazil [40], and Japan [41] in 2016. The global distribution of *K. pneumoniae* ST307 has been recorded in all continents except Antarctica and has been implicated in many nosocomial [42] and long-term care center outbreaks. Findings from Colombia [43], Texas, the United States [44], Argentina [45], and Italy [46] have also shown that the incidence of ST307 among CPKP has been rising over the years, even replacing other high-risk antibiotic-resistant strains, including ST258 in regions such as Italy and Colombia [43,46].

A recent study examined major nationwide nosocomial outbreaks of *bla*_OXA-181_ and *bla*_CTX-M-15_ within several South African provinces. *bla*_OXA-181_ was reported in 471 *K. pneumoniae* isolates belonging to ST307, isolated from 1,247 unique clinical isolates from a private laboratory network repository between 2014 and 2016. Bayesian evolution analysis showed that ST307 isolates have evolved into six clades with clades I–IV restricted to Texas in the United States and clade VI confined to South Africa. Clade V is, in contrast, globally distributed. Clade VI in South Africa originated in 2013 and developed into two distinct lineages during 2014, spreading over 15 months across 23 cities/towns in six provinces of South Africa [47].

Several carbapenemases have been identified in *K. pneumoniae* in South Africa. Lowe and colleagues (2019) reported a surge in *K. pneumoniae* producing OXA-48-like strains, contrary to two studies in 2016 that detected the predominance of *bla*_NDM-1_ compared to *bla*_OXA-48_ [47]. In 2016, Perovic and colleagues reported data from private sector laboratories in South Africa, where a high prevalence of *bla*_NDM-1_ was observed in Gauteng province compared to *bla*_OXA-48_ through an evaluation of 9029 Gram-negative ESKAPE (*K. pneumoniae*, *A. baumannii*, *P. aeruginosa*, *E. cloacae,* and *E. coli*) isolates [48]. A review in the same year (2016), assessing carbapenem resistance-reporting publications from January 2000 and May 2016, showed that most common carbapenem-resistant isolates were isolated in *Enterobacterales* such as *K. pneumoniae*, *E. cloacae*, and S. *marcescens* with NDM (*n* = 860) and OXA-48 (*n* = 584) among the most reported carbapenemases [49]. A recent report described another increment in *K. pneumoniae* producing OXA-48-like strains, where *bla*_OXA-48_ (65%), followed by *bla*_NDM-1_ (29%), was the most common carbapenemases detected [34], in contrast to the study findings where *bla*_OXA-181_ was dominant. *bla*_NDM-1_ to *bla*_OXA-48_ have also been identified in Gauteng and the Eastern Cape [48].

The isolates carried carbapenemase *bla*_OXA-181_, other β-lactamases, plasmid-mediated quinolone resistance (PMQR) genes, and other ARGs conferring resistance to several antibiotic classes (Table 3). This suggests the potential co-selection of resistance genes and, more importantly, HGT [50] via plasmids and other MGEs [51,52].

*bla*_CTX-M-15_ was located on IS*Ec9* in all isolates and most of the *bla_TEM-1B_* on IS*91* (Table 3). This globally observed pattern demonstrates the clonal and plasmid-mediated distribution of these ARGs within the same genetic context and on the same plasmid replicons in the same and different species. For example, IncF plasmids and IS*Ec9* have been shown to promote the global spread of *bla*_CTX-M-15_, alongside *bla*_OXA-10_, *aac(6′)Ib-cr*, and *bla*_TEM_ across different species [52]. The presence of these genes in similar genetic contexts was reported in another South African study describing 20 consecutive MDR *E. coli* isolates collected from a referral laboratory serving two secondary and three tertiary academic hospitals in Gauteng province. The study reported ARGs mediating resistance to fluoroquinolones, aminoglycosides, and tetracyclines in *E. coli* isolates alongside β-lactam resistance mediated by OXA, CTX-M, and TEM-1B. Many of these resistance determinants were located on contigs containing multiple plasmid replicons and bracketed by composite transposons (Tn*3*), various ISs, and class 1 integrons. IncF was the most common plasmid replicon. Class 1 integrons were the only integron type identified, and *bla*_CTX-M_ genes were frequently detected in IS*Ec9* [53], similar to the study findings.

The diverse mobilome consisting of different plasmid replicons (ColKP3, IncFIB(K), IncFII(K), IncFIB(pNDM-Mar), and IncX3) (Table 2) and insertion sequences (Table 3) intimates the potential mobility of this *bla_OXA-181_* by HGT among isolates. Furthermore, the global dominance of the IncF plasmid, an HGT-associated MGE, is verified in previous studies. For instance, Pedersen and colleagues confirmed the role of MGEs in the potential distribution of carbapenemases. They investigated the molecular epidemiology of CRE clinical samples (*n* = 45) collected by a private laboratory in Durban from hospitalized patients in 10 separate private hospitals between 2012 and 2013. Focusing on the carbapenem-resistance encoding determinants and their genetic support, they demonstrated that patterns of dissemination of CPE gens (*bla*_NDM-1_, *bla*_GES-5_, *bla*_OXA-232_, and *bla*_NDM-5_) via MGEs (e.g., integrons and insertion sequences) embedded in five different plasmids have been revealed to mediate their horizontal transfer, together with clonal transmission, between various *Enterobacterales* species [54]. Although a small number of CRKP isolates were obtained, this study adds to the limited knowledge on the genomic insights into the resistance mechanisms, mobile genetic support, and phylogenetic relationship of isolates resistant to last-resort antibiotics such as carbapenems in Africa. Future studies should consider using larger sample sizes from diverse geographical locations and settings to understand the resistance mechanisms and transmission dynamics of CREs.

## 5. Conclusions

OXA-181-producing *K. pneumoniae* belonging to ST307 was potentially endemic in the hospital ICU environment of a public hospital in KwaZulu-Natal, South Africa. Many ARGs and/or MGEs in different permutations and combinations present challenges to clinical management and infection prevention and control measures. This necessitates a CRE screening programme and strict infection prevention and control measures to detect and eliminate this endemic clone.

## Figures and Tables

**Table 1:** Patient demographics, antibiograms, sequence types, carbapenemase, other β-lactamases, and ARGs in *K. pneumoniae* isolates.

Isolate	Demographic Information						Resistome		Plasmid Incompatibility Group
	Patient	Gender	Date	Study Day	Antibiogram	ERIC Type	Other β-Lactamases	Other ARGs	
EC03607709	P2	Male	19 February 2020	Week 2	A	2	*bla*_CTX-M-15_, *bla*_OXA-1_, *bla*_SHV-106_, *bla*_TEM-1B_	*aac(6′)-Ib-cr*, *aph(3″)-Ib*, *aph(6)-Id*, *qnrS1*, *qnrB1*, *oqxB*, *oqxA*, *dfrA14*, *sul2*, *fosA*, *catB3*, *tet(A)*	ColKP3, IncFIB(K), IncFII(K), IncX3
EC03605938	P13	Male	18 February 2020	48 h	B	2	*bla*_CTX-M-15_, *bla*_OXA-1_, *bla*_SHV-106_, *bla*_TEM-1B_	*aac(6′)-Ib-cr*, *aph(3′)-VIa*, *armA*, *qnrS1*, *oqxB*, *oqxA*, *dfrA14*, *fosA*, *msr(E)*, *mph(E)*, *catB3*, *tet(A)*, *cmlA1*, *ARR-2*	ColKP3, IncFIB(K), IncFII(K), IncX3
EC03607707	P13	Male	19 February 2020	72 h	A	1	*bla*_CTX-M-15_, *bla*_OXA-1_, *bla*_SHV-106_, *bla*_TEM-1B_	*aac(6′)-Ib-cr*, *aph(3′)-VIa*, *armA*, *qnrS1*, *oqxB*, *oqxA*, *dfrA14*, *fosA*, *msr(E)*, *mph(E)*, *catB3*, *tet(A)*, *cmlA1*	ColKP3, IncFIB(K), IncFII(K), IncX3
EC03612985	P22	Female	24 February 2020	Discharge	C	5	*bla*_CTX-M-15_, *bla*_OXA-1_, *bla*_SHV-106_, *bla*_TEM-1C_	*aac(6′)-Ib-cr*, *aph(3″)-Ib*, *aph(6)-Id*, *qnrS1*, *qnrB1*, *oqxB*, *oqxA*, *dfrA14*, *sul2*, *fosA*, *catB3*, *tet(A)*	ColKP3, IncFIB(pNDM-Mar), IncX3
EC03623002	P30	Male	3 March 2020	48 h	A	1	*bla*_CTX-M-15_, *bla*_OXA-1_, *bla*_SHV-106_, *bla*_TEM-1B_	*aac(6′)-Ib-cr*, *aph(3″)-Ib*, *aph(6)- Id*, *qnrS1*, *qnrB1*, *oqxB*, *oqxA*, *dfrA14*, *sul2*, *fosA*, *catB3*, *tet(A)*	ColKP3, IncFIB(K), IncFII(K), IncX3
EC03629993	P30	Male	8 March 2020	Mero D4	A	2	*bla*_CTX-M-15_, *bla*_OXA-1_, *bla*_SHV-106_, *bla*_TEM-1B_	*aac(6′)-Ib-cr*, *aph(3″)-Ib*, *aph(6)-Id*, *qnrS1*, *qnrB1*, *oqxB*, *oqxA*, *dfrA14*, *sul2*, *fosA*, *catB3*, *tet(A)*	ColKP3, IncFIB(K), IncFII(K), IncX3
EC03632007	P30	Male	10 March 2020	Mero D6	A	4b	*bla*_CTX-M-15_, *bla*_OXA-1_, *bla*_SHV-106_, *bla*_TEM-1B_	*aac(6′)-Ib-cr*, *aph(3″)-Ib*, *aph(6)-Id*, *qnrS1*, *qnrB1*, *oqxB*, *oqxA*, *dfrA14*, *sul2*, *fosA*, *catB3*, *tet(A)*	ColKP3, IncFIB(K), IncFII(K)
EC03638959	P30	Male	14 March 2020	Week 2	A	4a	*bla*_CTX-M-15_, *bla*_OXA-1_, *bla*_SHV-106_, *bla*_TEM-1B_	*aac(6′)-Ib-cr*, *aph(3″)-Ib*, *aph(6)-Id*, *qnrS1*, *qnrB1*, *oqxB*, *oqxA*, *dfrA14*, *sul2*, *fosA*, *catB3*, *tet(A)*	ColKP3, IncFII(K)

Mero D6 = 6th day of meropenem therapy; Mero D4 = 4th day of meropenem therapy, A = AMP-AMC-TZP-CXM-CXM-AX-FOX-CTX-CAZ-FEP-ERTA-IPM-MEM-CIP-NIT-SXT; B = AMP-AMC-TZP-CXM-CXM-AX-FOX-CTX-CAZ-FEP-ERTA-MEM-CIP-NIT-SXT; C = AMP-AMC-TZP-CXM-CXM-AX-FOX-CTX-CAZ-FEP-ERTA-MEM-GEN-CIP-NIT-SXT.

**Table 2:** Plasmid replicon types, class 1 integrons, and gene cassettes found in *K. pneumoniae* isolates.

Isolate	Plasmids Replicons	pMLST	Integron Class	Integron	Cassette Arrays					
					GC1 ^a^	GC2	GC3	GC4	GC5	GC6
EC03607709	ColKP3, IncFIB(K), IncFII(K), IncX3	[K7:A-:B-]	Integron integrase IntI1	In191	*dfrA14*	— ^b^	—	—	—	—
EC03638959	ColKP3, IncFII(K)	[K7:A-:B-]	Integron integrase IntI1	In191	*dfrA14*	—	—	—	—	—
EC03632007	ColKP3, IncFIB(K), IncFII(K)	[K7:A-:B-]	ND	ND	*dfrA14*	—	—	—	—	—
EC03629993	ColKP3, IncFIB(K), IncFII(K), IncX3	[K7:A-:B-]	Integron integrase IntI1	In191	*dfrA14*	—	—	—	—	—
EC03607707	ColKP3, IncFIB(K), IncFII(K), IncX3	[K7:A-:B-]	Integron integrase IntI1	In191	*dfrA14*	—	—	—	—	—
EC03623002	ColKP3, IncFIB(K), IncFII(K), IncX3	[K7:A-:B-]	Integron integrase IntI1	In191	*dfrA14*	—	—	—	—	—
EC03605938	ColKP3, IncFIB(K), IncFII(K), IncX3	[K7:A-:B-]	Integron integrase IntI1	In191	*dfrA14*	—	—	—	—	—
EC03612985	ColKP3, IncFIB(pNDM-Mar), IncX3	[F-:A-:B-]	Integron integrase IntI1	In27	*dfrA12*	*gcuF*	*aadA2*	—	—	—

Note: ^a^ GC denotes gene cassettes. ^b^ denotes the missing cassette arrays due to draft genomic sequences that were fragmented during the sequencing and assembling process into different contigs.

**Table 3:** ARGs and MGEs in synteny and their plasmidic/chromosomal locations.

Isolate	Contig	Synteny of Resistance Genes and MGEs	Plasmid/Chromosomal Sequence with the Closest Nucleotide Homology (Accession Number)
EC03607709	49	*bla*_OXA-181_:*EreA*::IS*Kra4* (IS*Kpn19*):recombinase::::recombinase:*QnrS1*:transposase	*E. coli* p010_B-OXA181 (99.98%), accession (CP048332.1) (plasmid)
	34	IS*5*:IS*5075* (IS*110*)::*Sul2*:*aph(3″)-Ib*:*aph(6)-Id*:IS*91*:*TEM-1B*:recombinase::IS*1380* (IS*Ec9*):*bla*_CTX-M-15_::Tn*3* family:IS*6* (IS*6100*)	p72_FIBkpn (100%), accession (CP034282.1) (plasmid)
	54	*CatB3:bla*_OXA-1_ *:aac(6′)-Ib-cr5*	*E. coli pYJ3-a* DNA (100%), accession (AP023228.1) (plasmid)
	15	::::*bla*_SHV-10*6*_::::	*K.p* F16KP0053 chromosome (100%), accession (CP052727.1)
	50	IS*6* (IS*6100*)::*dfrA14*:IntI1	*K.p* pB16KP0177-1 (100%), accession (CP052525.1) (plasmid)
EC03638959	95	transposase:*QnrS1*:recombinase::::recombinase:IS*Kra4* (IS*Kpn19*)::*EreA*:*bla*_OXA-181_	*E. coli* p010_B-OXA181 (99.98%), accession (CP048332.1) (plasmid)
	98	*bla_TEM-1B_*:recombinase::IS*1380* (IS*Ec9*):*bla*_CTX-M-15_::Tn*3* family:IS*6* (IS*6100*)	p72_FIBkpn (100%), accession (CP034282.1) (plasmid)
	109	*aac(6′)-Ib-cr5*:*bla*_OXA-1_:*CatB3*	*E. coli pYJ3-a* DNA (100%), accession (AP023228.1) (plasmid)
	24	::::*bla*_SHV-10*6*_::::	*K.p* F16KP0053 chromosome (100%), accession (CP052727.1)
	102	*dfrA14*::IS*6* (IS*6100*)	*K.p* pB16KP0177-1 (100%), accession (CP052525.1) (plasmid)
EC03632007	93	*bla*_OXA-181_:*EreA*::IS*Kra4* (IS*Kpn19*):recombinase::::recombinase:*QnrS1*:transposase	*E. coli* p010_B-OXA181 (99.98%), accession (CP048332.1) (plasmid)
	96	Tn*3* family::*bla*_CTX-M-15_:IS*1380* (IS*Ec9*)::recombinase:TEM-1B	p72_FIBkpn (100%), accession (CP034282.1) (plasmid)
	112	*CatB3*:*bla*_OXA-1:_*aac(6′)-Ib-cr5*	*E. coli pYJ3-a* DNA (100%), accession (AP023228.1) (plasmid)
	23	::::*bla*_SHV-106_::::	*K.p* F16KP0053 chromosome (100%), accession (CP052727.1)
	101	*dfrA14*::IS*6* (IS*6100*)	*K.p* pB16KP0177-1 (100%), accession (CP052525.1) (plasmid)
EC03629993	47	*bla*_OXA-181:_*EreA*::IS*Kra4* (IS*Kpn19*):recombinase::::recombinase:*QnrS1*:transposase	*E. coli* p010_B-OXA181 (99.98%), accession (CP048332.1) (plasmid)
	35	IS*5*:IS*5075* (IS*110*)::*Sul2*:*aph(3″)-Ib:aph(6)-Id*:IS*91*:TEM-1B:recombinase::IS*1380* (IS*Ec9*):*bla*_CTX-M-15_::Tn*3* family:IS*6* (IS*6100*)	p72_FIBkpn (100%), accession (CP034282.1) (plasmid)
	55	*aac(6′)-Ib-cr5*:*bla*_OXA-1:_*CatB3*	*E. coli pYJ3-a* DNA (100%), accession (AP023228.1) (plasmid)
	16	::::*bla*_SHV-106_::::	*K.p* F16KP0053 chromosome (100%), accession (CP052727.1)
	49	IntI1:*dfrA14*::IS*6* (IS*6100*)	*K.p* pB16KP0177-1 (100%), accession (CP052525.1) (plasmid)
EC03607707	50	*bla*_OXA-181_:*EreA*::IS*Kra4* (IS*Kpn19*):recombinase::::recombinase:*QnrS1*:transposase	*E. coli* p010_B-OXA181 (99.98%), accession (CP048332.1) (plasmid)
	36	IS*6* (IS*6100*):Tn*3* family*::bla*_CTX-M-15_:IS*1380* (IS*Ec9*)::recombinase:TEM-1B:IS*91*:*aph(6)-Id:aph(3″)-Ib:Sul2*::IS*5075* (IS*110*):IS*5*	p72_FIBkpn (100%), accession (CP034282.1) (plasmid)
	56	*CatB3*:*bla*_OXA-1_:*aac(6′)-Ib-cr5*	*E. coli pYJ3-a* DNA (100%), accession (AP023228.1) (plasmid)
	13	::::*bla*_SHV-106_::::	*K.p* F16KP0053 chromosome (100%), accession (CP052727.1)
	52	IS*6*(IS*6100*)::*dfrA14*:IntI1	*K.p* pB16KP0177-1 (100%), accession (CP052525.1) (plasmid)
EC03623002	45	*bla*_OXA-181_:*EreA*::IS*Kra4* (IS*Kpn19*):recombinase::::recombinase:*QnrS1*:transposase	*E. coli* p010_B-OXA181 (99.98%), accession (CP048332.1) (plasmid)
	32	IS*5*:IS*5075* (IS*110*)::*Sul2*:*aph(3″)-Ib*:*aph(6)-Id*:IS*91*:TEM-1B:recombinase::IS*1380* (IS*Ec9*):*bla*_CTX-M-1*5*_::Tn*3* family:IS*6* (IS*6100*)	p72_FIBkpn (100%), accession (CP034282.1) (plasmid)
	54	*aac(6′)-Ib-cr5*:*bla*_OXA-1_:*CatB3*	*E. coli pYJ3-a* DNA (100%), accession (AP023228.1) (plasmid)
	16	::::*bla*_SHV_-_106_::::	*K.p* F16KP0053 chromosome (100%), accession (CP052727.1)
	47	IntI1:*dfrA14*::IS*6* (IS*6100)*	*K.p* pB16KP0177-1 (100%), accession (CP052525.1) (plasmid)
EC03605938	48	transposase:*QnrS1*:recombinase::::recombinase:IS*Kra4* (IS*Kpn19*)::*EreA*:*bla*_OXA-181_	*E. coli* p010_B-OXA181 (99.98%), accession (CP048332.1) (plasmid)
	34	IS*6* (IS*6100*):Tn*3* family::*bla*_CTX-M-15*:*_IS*1380* (IS*Ec9*)::recombinase:TEM-1B:IS*91*:*aph(6)-Id:aph(3″)-Ib:Sul2*::IS*5075* (IS*110*):IS*5*	p72_FIBkpn (100%), accession (CP034282.1) (plasmid)
	53	*aac(6′)-Ib-cr5:bla*_OXA-1_ *:CatB3*	*E. coli pYJ3-a DNA* (100%), accession (AP023228.1) (plasmid)
	14	::::*bla*_SHV-106_::::	*K.p* F16KP0053 chromosome (100%), accession (CP052727.1)
	50	IntI1*:dfrA14*::IS*6* (IS*6100*)	*K.p* pB16KP0177-1 (100%), accession (CP052525.1) (plasmid)
EC03612985	48	Tn*3-like* IS*3000*:*bla*_OXA-181_:*EreA*::IS*Kra4* (IS*Kpn19*):recombinase::::recombinase:*QnrS1*:transposase	*E. coli* p010_B-OXA181 (99.98%), accession (CP048332.1) (plasmid)
	54	*::bla*_CTX-M-15_:IS*1380* (IS*Ec9*)::recombinase:blaTEM-1C::	*E. coli* str. 473 pRCS52 (99.9%), accession (LO017736.1) (plasmid)
	61	*aac(6′)-Ib-cr5*:*bla*_OXA-1_:*CatB3*	*E. coli pYJ3-a DNA* (100%), accession (AP023228.1) (plasmid)
	2	::::*bla*_SHV-106_::::	*K.p* F16KP0053 chromosome (100%), accession (CP052727.1)
	49	recombinase::IntI1:*dfrA12:gcuF:aadA2::Sul1*::::::::IS*6* (IS*6100*)::::*Mph(A*)	*K.p* plasmid unnamed1 (99.99%), accession (CP060050.1) (plasmid)
	59	IS*3*:*aac(3)-IIa*	*K.p* pMS14393B (100%), accession (CP054305.1) (plasmid)

## Data Availability

All analyzed data have been included in the manuscript. The nucleotide sequences of the eight isolates analyzed in this study were deposited in the NCBI GenBank database in the Bioproject number (PRJNA674742): under the Accession numbers; JADKPL000000000.1, JADOEH000000000.1, JADOEG000000000.1, JADKPK000000000.1, JADKPJ000000000.1, JADKPI000000000.1, JADKPH000000000.1, and JADKPG000000000.1.

## Supplementary Materials

The following are available online at https://www.mdpi.com/article/10.3390/genes12070951/s1, Table S1: Comparison of *K. pneumoniae* genomes with South African genomes, downloaded from NCBI and PATRIC servers; Table S2: Patients’ demographics; Table S3: Antibiotic susceptibility characteristics (MICs) of the carbapenemase-producing *K. pneumoniae* (CPKP) isolates collected from patients in an intensive care unit (ICU); Table S4: General genomic features of eight sequenced *K. pneumoniae* isolates; Table S5: Virulence analysis of the *K. pneumoniae* isolates showing the virulence genes repertoire (all isolates carried the same repertoire of 65 genes).
